# A Haptic-Driven Serious Game for Cognitive Stimulation and Visual Impairment Mitigation in Older Adults Based on the Design-Play-Experience Framework: Cross-Sectional Mixed Methods Pilot Study

**DOI:** 10.2196/86290

**Published:** 2026-01-30

**Authors:** Xin Huang, Nazlena Mohamad Ali, Shafrida Sahrani, Yue Zhang

**Affiliations:** 1 Institute of Visual Informatics (IVI) Universiti Kebangsaan Malaysia Selangor Malaysia

**Keywords:** serious games, haptic feedback, older adults, cognitive abilities, visual impairment, DPE framework, Design-Play-Experience, pilot study

## Abstract

**Background:**

In the context of global aging, cognitive decline among older adults has become a prevalent issue, significantly impacting their daily lives. Serious games have demonstrated potential in enhancing cognitive abilities in this population. However, most existing serious games designed for older adults rely heavily on visual interfaces, which are often potentially detrimental for those with pre-existing visual impairments.

**Objective:**

This study had two primary objectives: (1) to design a theoretical prototype for a haptic-driven serious game for older adults based on the Design-Play-Experience (DPE) framework, aiming to enhance cognitive abilities, including attention, logical reasoning, and decision-making while simultaneously mitigating challenges associated with visual impairment, and (2) to conduct a pilot study evaluating the prototype’s usability, accessibility, and user experience within the target population.

**Methods:**

We used a cross-sectional, mixed methods pilot study with a single-group observational design, comprising a theoretical design and a pilot user study. First, the DPE framework was systematically applied to develop a game prototype by integrating haptic feedback technology (using built-in smartphone vibration motors) across its 3 core dimensions: design (haptic symbol system, accessible interface), play (dynamic difficulty adjustment), and experience (emotional engagement). Subsequently, a pilot study was conducted with 10 older adults recruited via convenience sampling (mean age 62.9, SD 3.35 years; 5 male, 5 female; all with self-reported mild visual impairments, such as presbyopia). Following interaction with the prototype, data were collected remotely using the System Usability Scale (SUS) and semistructured interviews administered via videoconferencing. Quantitative data from the SUS were analyzed using descriptive statistics, while qualitative data from the interviews were processed using thematic analysis.

**Results:**

Pilot user studies showed that the game prototype had good usability, with an average SUS score of 89.5 (SD 2.72; 95% CI 87.6-91.4), which is considered “excellent.” Thematic analysis of the interviews revealed three significant themes. The first theme was intuitive haptic feedback, which reflected that participants were able to quickly grasp and value the vibrational cues used to identify cards. The second theme was based on reduced eye strain, in which the combination of large fonts, high-contrast interfaces, and haptic feedback was praised for its effectiveness in relieving eye strain. The third theme was simplicity, where the simplified card game mechanics were considered both fun and challenging.

**Conclusions:**

This study developed and validated a haptic, serious game for older adults. Its innovation lies in the systematic application of the DPE framework to achieve “haptic substitution for vision,” which differs from previous research that focused on general immersive experiences. The main contribution of this study is providing a reusable design blueprint for creating easy-to-use cognitive training tools. These findings have practical implications in the real world, providing a feasible approach for deploying low visual load interventions in communities and care facilities.

## Introduction

By 2050, two-thirds of the global population aged 60 years and older will live in low- and middle-income countries [1]. The population structure is undergoing a significant aging transition, and the most common cognitive diseases in older adults include mild cognitive impairment [2], dementia [3], and Parkinson disease [4]. Mild cognitive impairment is a cognitive disorder between dementia and health [5]. At this time, the patient’s memory and cognitive function have already had problems, and one or more cognitive functions have declined, but the patient’s daily life will not be significantly affected [6,7]. Dementia affects 36 million people worldwide [8]. Dementia is closely associated with age (aging), with nearly half of the population aged 85 years and older developing dementia [9]. The most common type of dementia is Alzheimer disease. The typical initial symptom is memory loss. Parkinson disease usually occurs in people aged 60 years and older. It is a chronic neurodegenerative disease that affects the central nervous system, with the most obvious early symptoms being tremors, limb stiffness, decreased movement, and gait abnormalities [10]. Parkinson disease may also cause cognitive and behavioral problems.

Serious games have had some success in helping older adults recover cognitive abilities [11]. Serious games are a set of solutions designed to make a range of rehabilitation courses more engaging and less boring [12]. MINWii (Samuel Benveniste, Pierre Jouvelot, and Renaud Pequignot) is a new serious video game tailored for people with Alzheimer disease and dementia, in which older gamers use Wiimotes to improvise or play predefined songs on a virtual keyboard displayed on the screen [13]. However, vision impairments in older adults may affect their interaction with serious games [14]. Older adults often experience sensory changes that affect their interaction with digital displays and games. These changes include decreased vision, impaired dark adaptation, reduced contrast sensitivity, limited visual accommodation range, reduced color sensitivity, and increased sensitivity to glare [14]. These impairments can make it difficult for older adults to perceive small elements on a screen, read text, or navigate complex interfaces [15]. The older adults said that the mobile phone screen was too small when interacting, which shows that the limitations of digital tools themselves are also one of the factors that affect the older adults’ interaction with serious games [16]. These studies show that, in addition to the decline of the older adult’s own sensory organs over time, serious games designed for older adults do not fully consider the older adult’s user experience.

The addition of haptic feedback technology further expands the impact of serious games [17] and can reduce the visual damage caused by long-term serious game playing by older adults. The haptic experience provides a new way of perception for older adults, thereby enhancing the user’s cognitive ability. Haptic technology is a sensory feedback technology that simulates haptic perception through force, vibration, or motion. It captures the user’s movements and interaction information through sensors and generates corresponding haptic feedback through actuators, allowing users to feel real physical interactions in a virtual environment [17]. Haptic feedback technology provides an immersive experience for older adults by combining their sense of touch with what they see, hear, or interact with [18]. At the same time, serious games combined with haptic feedback technology have shown the potential to significantly improve cognitive abilities [19]. For example, in mobile serious games, the interaction method developed by Deng et al [20] that combines eye tracking with haptic feedback also significantly improves the user’s operation accuracy and task completion rate. By providing haptic feedback on mobile devices, the user’s error rate in interactive tasks is significantly reduced, indicating that serious games combined with haptic feedback can effectively enhance the user’s understanding and control of cognitive abilities [20]. Silva et al [21] reported a new haptic device under development, mainly for older adults, to stimulate and quantify the response of the older adults’ nervous system through serious games.

The existing combination of serious games and haptic feedback technology mainly focuses on enhancing interactive immersion. Although it has improved the cognitive ability of some older people, there are few system frameworks specifically for older adults with visual impairment. There is a lack of deep integration of cognitive training and haptic feedback. Its design is mostly oriented to general users and lacks targeted adaptation for the visual and haptic coordination needs of older adults. There are 2 major limitations in the current research on the use of serious games by older adults, one being the problem of visual dependence. Most serious games rely on complex visual interfaces, which aggravate the interaction barriers caused by vision decline in older adults. The second is the one-sidedness of haptic feedback design. Although haptic technology can enhance immersion through force feedback, its application focuses on general scenarios and lacks a system framework for the cognitive decline and sensory coordination needs of older adults (such as “haptic substitution for vision”).

To address these shortcomings, this study uses the Design-Play-Experience (DPE) framework [22] as its theoretical basis to develop a prototype of a haptic-driven serious card game. The DPE framework provides a structured approach to integrate technology, game mechanics, and user experience, making it well-suited for systematically incorporating haptic feedback to achieve specific cognitive and accessibility goals. The final prototype embodies this integration (1) in the design dimension, through a haptic symbol system and a high contrast interface; (2) in the play dimension, through haptic cues to dynamically adjust the game difficulty; and (3) in the experience dimension, through haptic rewards to enhance emotional engagement. The objective of this study is to develop a haptic feedback serious card game theoretical prototype based on the DPE framework to reduce the visual burden of older adults and improve their cognitive ability. Second, to conduct a pilot study evaluating the prototype’s usability, accessibility, and user experience within the target population. The contribution of this study is to combine haptic feedback with the card game mechanism and provide a reusable design blueprint for the design of older adult–friendly serious games through the system mapping of the DPE framework.

## Methods

### Quantitative Analysis

#### Inclusion and Exclusion

The inclusion criteria for participants were (1) age ≥60 years; (2) self-reported mild visual impairment, such as presbyopia; (3) basic smartphone usage skills; and (4) willingness to sign an informed consent form. The exclusion criteria included (1) being diagnosed with severe cognitive impairment, such as clinically diagnosed dementia, and (2) having severe hearing loss or motor impairment that significantly affects haptic perception or device operation.

#### Participant Characteristics

Participants had a mean age of 62.9 (SD 3.35) years, with an age range of 60-71 years, and comprised 5 males and 5 females. All participants reported having mild visual impairment, such as presbyopia, and possessed basic smartphone operation skills.

#### Sampling Procedures

Participants were recruited from Dangtu Old Age University, using convenience sampling [23]. Recruitment and data collection were conducted remotely from June 1 to July 1, 2025. The research team also collaborated with the institution’s administration to disseminate recruitment information through employee referrals. Interested potential seniors contacted the research team by phone, and those who passed the initial screening were formally enrolled.

#### Sample Size, Power, and Precision

As a pilot study, the sample size (N=10) was determined primarily based on feasibility considerations rather than statistical power calculations. This sample size is typical for pilot studies and aims to provide preliminary data and process validation for subsequent large-scale efficacy trials.

#### Measures and Covariates

The primary outcome measure was usability of the game prototype, measured using the System Usability Scale (SUS). The scale’s total score ranges from 0 to 100. The secondary outcome measure includes accessibility and user experience. In covariates and confounding factors, participants’ baseline cognitive level, severity of visual impairment, technical experience, and age were recorded as potential confounding factors and qualitatively documented during interviews via background questions.

#### Data Collection

Quantitative data were collected through online survey platforms, such as Wenjuanxing [24]. Participants completed a SUS [25] online after interacting with the game prototype.

#### Quality of Measurements

To ensure data quality, all data collection procedures were standardized. Specifically, all participants used the same game prototype and completed the same SUS. Furthermore, a consistent interview process was followed, with semistructured interviews [26] conducted by the same researcher after participants had finished experiencing the game, using interview guidelines to ensure consistency across all interviews. The SUS [25], the primary quantitative tool in this study, has had its reliability and validity extensively confirmed in previous research [27,28].

#### Instrumentation

The main tools used in this study were the SUS and a haptic game prototype developed based on the DPE framework. This prototype runs on an Android smartphone and uses the device’s built-in linear resonant motor to provide structured haptic feedback.

#### Masking

Given that this study used a single-group observational design aimed at directly obtaining users’ subjective feedback on the prototype, no masking was applied to participants or researchers.

#### Psychometrics

This study used the widely validated SUS, whose reliability and validity are supported by literature [27]. SUS has demonstrated excellent psychometric properties, including good reliability (eg, high internal consistency, Cronbach alpha coefficient typically above 0.85, and test-retest reliability of approximately 0.80) and strong validity (convergent validity, discriminant validity, and construct validity) [25,27,28]. This study directly reports the SUS scores observed in the current sample.

#### Conditions and Design

This study uses a nonexperimental observational design, specifically a single-group, cross-sectional study. Reporting of the study was performed in accordance with the JARS (Journal Article Reporting Standards) guidelines [29].

#### Data Diagnostics

The study conducted diagnostic checks on the data. All 10 participants completed the entire study process, and there were no missing data for either the primary or secondary outcome measures. Therefore, no imputation was performed. Given the small sample size, the distribution of SUS scores was examined. No extreme outliers requiring intervention were found, and no data transformation was performed.

#### Analytic Strategy

Quantitative data analysis used descriptive statistics. The total score of the SUS was described using the mean and SD, and 95% CIs based on a *t* test distribution were calculated to provide more conservative interval estimates for small samples. All analyses aimed to assess the initial usability of the game prototype.

### Qualitative Analysis

#### Research Design Overview

The qualitative portion of this study used a thematic analysis [30] approach to explore older users’ experiences, perceived accessibility, and emotional engagement with haptic-driven game prototypes. This design was well-suited for gathering detailed participant-centered perspectives from semistructured interviews.

#### Study Participants or Data Sources

The qualitative data came from the same 10 participants (mean age of 62.9, SD 3.35 years; 5 male and 5 female) as the quantitative data. The data were in the form of audio recordings of semistructured interviews with each participant and their verbatim transcripts.

#### Participant Recruitment

The recruitment process for participants was the same as that for the quantitative part. Convenience sampling was used to recruit from Dangtu Old Age University. The research team collaborated with the institution to make initial contact, and eligible volunteers were enrolled after confirmation by phone. All participants in the quantitative assessment completed subsequent qualitative interviews to ensure the continuity of data sources.

### Data Collection

This study collected qualitative data through semistructured interviews conducted using videoconferencing software, such as Tencent Meeting [31]. We developed an interview guideline to explore participants’ experiences in three areas: (1) the intuitiveness of haptic feedback, (2) the impact of haptic feedback on alleviating visual fatigue, and (3) the overall appeal and engagement of the game. This approach ensured comprehensive data collection. All interviews were conducted immediately after participants completed the game experience and SUSs to ensure the freshness of the experience. Data collection continued until thematic saturation was reached. All interviews were recorded with the participants’ consent and transcribed by researchers for analysis. All interviews were recorded with the participants’ consent and transcribed by researchers for analysis.

### Ethical Considerations

The study was approved by the ethics committee before it was carried out, and it strictly adhered to the ethical guidelines for research involving human participants. The study protocol, including the data collection methods, was considered to comply with the ethical standards for research involving human participants (approval reference UKM.IVI.600/8/1-P136397). Written informed consent was obtained from all participants before participating in the study. The consent form clearly outlined the purpose, procedures, risks, benefits, and voluntary nature of participating in the study. Participants were informed of their right to withdraw at any time without penalty. For secondary data analysis, the original consent form explicitly allowed the use of deidentified data for research purposes without the need for additional consent. All participant data were anonymized and deidentified during collection and analysis. Personally identifiable information was removed from survey responses, and data were securely stored on a password-protected server accessible only to the research team. No identifiable information will be shared with external parties or institutions. Participants received no monetary or nonmonetary compensation for their participation in this study. Their contribution was voluntary and motivated by the potential societal benefits of the research. No identification of individual participants in any images of the paper or supplementary material is possible. All data reported were aggregated and anonymized to ensure participant confidentiality. This study conformed to the tenets of the Declaration of Helsinki and adhered to the ethical standards outlined by JMIR for research involving human participants.

## Results

### Participant Flow and Demographics

All 10 recruited older participants (5 male and 5 female; mean age 62.9, SD 3.35; 60-71 years) completed the entire research process, including interactive games, the SUS questionnaire, and interviews. No data were missing for either the primary or secondary outcome measures.

### Quantitative Analysis

Quantitative analysis of the pilot study showed that the haptic game prototype received a very high usability rating. The mean score on the SUS was 89.5 (SD 2.72; 95% CI 87.6-91.4). According to the scale’s evaluation criteria, this score falls into the “excellent” category, clearly indicating that the prototype possesses both high usability and high acceptability among the target older adults (Table 1).

**Table 1:** System Usability Scale (SUS) scores from a cross-sectional pilot study evaluating a haptic-driven serious game prototype for cognitive stimulation and visual impairment mitigation in older adults.

Metric	Value
SUS score, mean (SD; 95% CI)	89.5 (2.72; 87.6-91.4)
Adjective rating	Excellent
Score range	85.0-92.5

### Qualitative Analysis

After conducting a thematic analysis of interviews, researchers identified three prominent themes regarding user experience and usability:

1. Intuitive haptic feedback: Participants quickly grasped and highly valued the vibrational cues used to identify cards. This haptic feedback was considered a natural and effective alternative to constant visual confirmation. One participant stated, “I quickly learned what the different vibrations represented; I didn’t have to constantly stare at the screen to remember what cards I was holding.”
2. Effective reduction of eye strain: The combination of haptic feedback and a high-contrast, large-font visual interface significantly reduced eye strain and made gameplay more comfortable. One participant commented, “The large font and vibrational cues combined made my eyes much more comfortable.”
3. Simplicity: The simplified game mechanics (a simplified 7-card Doudizhu) were considered both fun and challenging, lowering the learning curve while maintaining player engagement through a dynamic difficulty adjustment system. One participant said, “It’s easy to pick up, but still requires thought. It’s fun without being frustrating.”

### A Theoretical Prototype of Serious Card Games Based on the DPE Framework

Table 2 is a mapping table of the DPE framework in game design. It comprehensively elaborates on the DPE framework for serious games aimed at older adults from 3 vertical dimensions (design, play, and experience) and 4 horizontal dimensions (learning, storytelling, gameplay, and user experience). The paper designs a theoretical prototype of a serious game with haptic feedback, aiming to improve the cognitive abilities of older adults and alleviate their visual impairments. In the design dimension, we created a haptic symbol system to reduce reliance on vision. In the gameplay dimension, we designed haptic cues to facilitate dynamic adjustment of game difficulty, and in the experience dimension, we added haptic rewards to enhance the player’s emotional engagement.

**Table 2:** Prototype structure mapping for a haptic-driven serious card game based on the Design-Play-Experience (DPE) framework.

Core Pillar	Design	Play	Experience
Learning	Content: Target cognitive abilities, such as attention, thinking, decision-making, and logical reasoning. Teaching methods: digital cognition, simple mathematical operations, and decision-making.	Teaching: Roles: Players play community members and play card games with virtual opponents. Setting: Community environment, build the community by winning games. Narrative: Earn coins by winning games, and build the community until the community is complete.	Learning: Through gaming activities, older adults can practice and improve their cognitive abilities. Self-efficacy: By successfully completing tasks through gaming, older adults can enhance their sense of self-efficacy and improve their self-confidence.
Storytelling	Roles: Players play community members and play card games with virtual opponents. Setting: Community environment where players build the community by winning games. Narrative: Earn coins by winning games, and build the community until the community is complete.	Storytelling: Players win coins and build communities by winning games. Interactive narrative: The storyline can have different development paths based on the player’s choices and performance.	Story: Provide a rich story experience through the game storyline to increase the appeal of the game.
Gameplay	Mechanics: A simplified version of Doudizhu, with only 7 cards, smaller than playing cards. Haptic feedback prompts, identifying the size of the card based on the number of vibrations (card 1, slight vibration 1 time, and so on).	Dynamics: A smooth gameplay flow ensures a cohesive and engaging experience. Difficulty Adjustment: Haptic feedback automatically adjusts the game difficulty based on player performance, maintaining the game’s challenge and fun.	Emotion: Design game elements that evoke positive emotional experiences, such as a sense of accomplishment and satisfaction. Provide unique haptic feedback as a reward when players achieve something or win, enhancing emotional engagement and a sense of accomplishment.
User experience	User interface: Large fonts, high contrast, and a simple operation interface are suitable for older adults. Navigation: Clear navigation and instructions help older adults easily find the required functions.	Interactivity: Encourage players to interact with virtual opponents through game tasks. Feedback interaction: Players’ actions will receive immediate feedback, enhancing their sense of participation.	Engagement: Design game elements that can attract players to continue to participate, such as achievement systems, reward mechanisms, etc. Enhance players’ sense of belonging and community through the game community.

### Game Prototype Interface Display

This study follows a prototype based on the DPE framework and systematically develops the visual and interactive design of the “Old Friends” game. We follow the WCAG 2.1 [32] accessibility standard and are explicitly committed to minimizing the visual and cognitive burden on older users throughout the design process.

**Figure 1:**
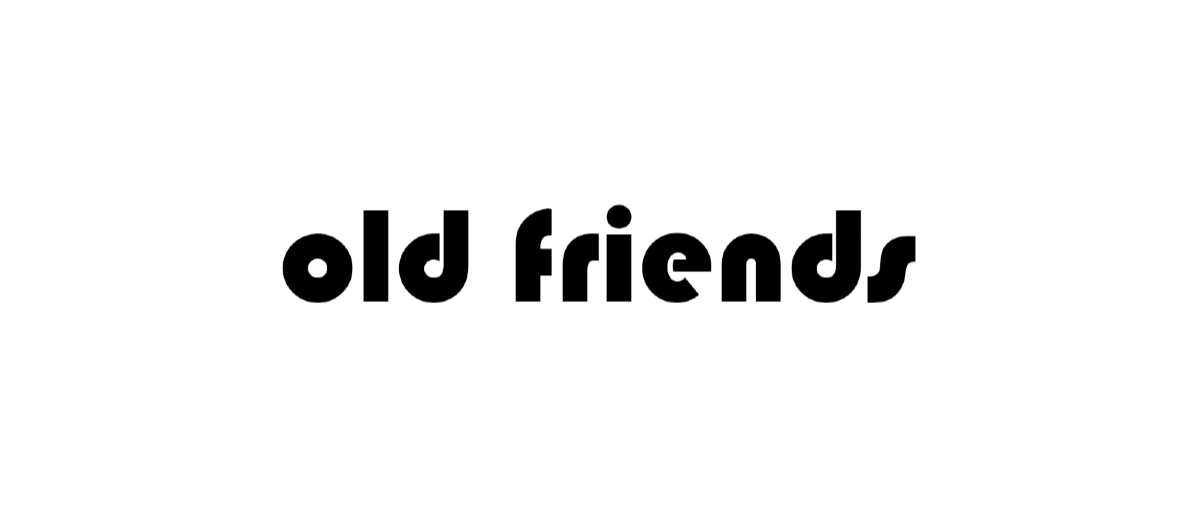
Logo of the “Old Friends” serious card game, compliant with WCAG (Web Content Accessibility Guidelines) 2.1 standards and embodying high-contrast visual identity principles.

The interface design intentionally used large, clear, and legible fonts (˃18 pt) and high contrast (˃ 4.5:1), consistently applied to all core visual components, including the game logo, card elements, login paper, main game, win or lose, and homeland interface. These choices directly supported the user experience goal emphasized in the design framework, focusing on clarity and usability. A key outcome of this process was the “Old Friends” logo (Figure 1). Its design exemplifies how accessibility principles translate into tangible visual identity. We chose the Bauhaus 93 font for its high legibility and clear glyphs, and simultaneously, we used a simple black-on-white color scheme to ensure maximum readability in accordance with the “minimum visual load principle.” However, this functional clarity is complemented by carefully designed emotional elements. The name “Old Friends” and its warm, friendly presentation aim to resonate with the project’s core objectives, emotional support, and social interaction, as detailed in our “Storytelling” and “Experience” dimensions. We believe this semantic and visual consistency enhances the emotional connection between users and the game, thereby increasing user engagement and strengthening community awareness. Therefore, the logo is not merely an aesthetic element but a reflection of a design philosophy that perfectly blends technological usability with an empathetic, goal-oriented narrative design.

**Figure 2:**
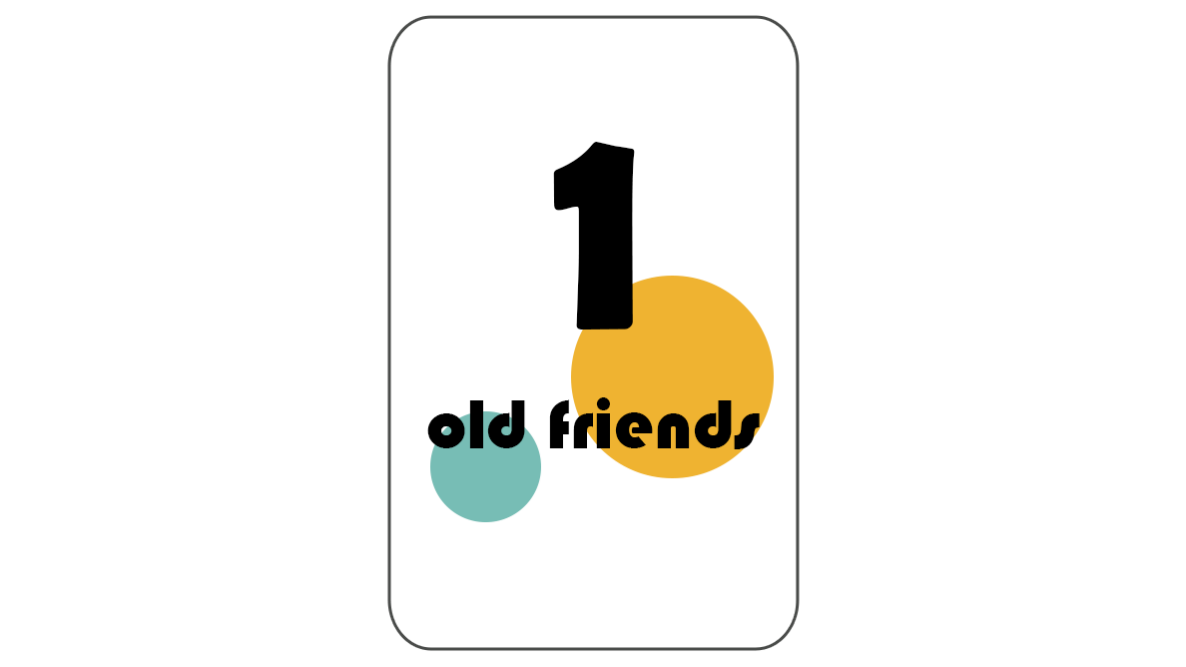
Game card interface of the “Old Friends” serious card game.

Figure 2 shows the final card design, developed through iterative iterations while consistently prioritizing senior friendliness. A core design decision was to use the clear and legible Berlin Sans Demi font to represent the numbers 1-7 at an oversized 120-point font size to ensure quick recognition. This design, combined with a high-contrast black-on-white scheme, forms the functional foundation of the card. Subsequently, the designers consciously layered various aesthetic elements on top of this easily understood foundation. Light blue and medium yellow circular decorations were introduced, adding visual interest and warmth without compromising clarity. The “Old Friends” logo, with its coordinated typography, visually and thematically unifies the entire component. This fusion of clear functionality and warm aesthetics was a thoughtful choice, designed to directly support the research goals of “emotional support” and “social interaction” by making the interface more appealing and personal for older users.

**Figure 3:**
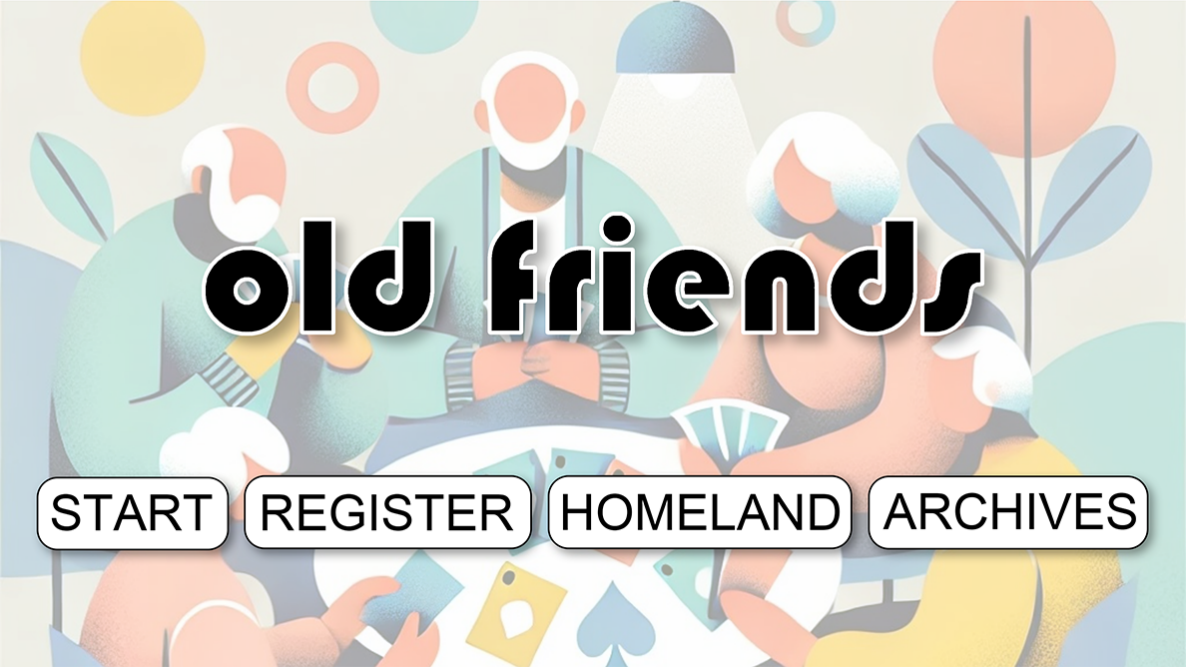
Login page interface of the “Old Friends” serious card game.

The login screen (Figure 3) is designed to immediately create a user-friendly atmosphere. To this end, we used a soft, light background, accented with soft yellow, orange, and blue circular graphic elements. This color scheme was deliberately chosen to create a warm and vibrant atmosphere without being overly stimulating to the senses, directly adhering to the “minimum visual load principle” and minimizing strong contrasts in unnecessary areas. Functional clarity is paramount. The central title, “Old Friends,” uses a high-contrast 60-point Bauhaus 93 font to ensure users can immediately recognize the brand. Similarly, the key operation buttons at the bottom of the screen (Start, Register, Homeland, Archives) use a standard, clear, and legible 18-point Arial font. They are arranged with ample spacing on a white background, a carefully designed interaction method intended to prevent accidental clicks and guide users easily into the game. Beyond functionality, the illustration of older people playing card games in the background also carries significant narrative meaning. It was included to visually convey the core themes of “emotional support” and “social interaction” from the outset, preparing users for a positive social experience and fostering a direct emotional connection with the game’s objectives.

**Figure 4:**
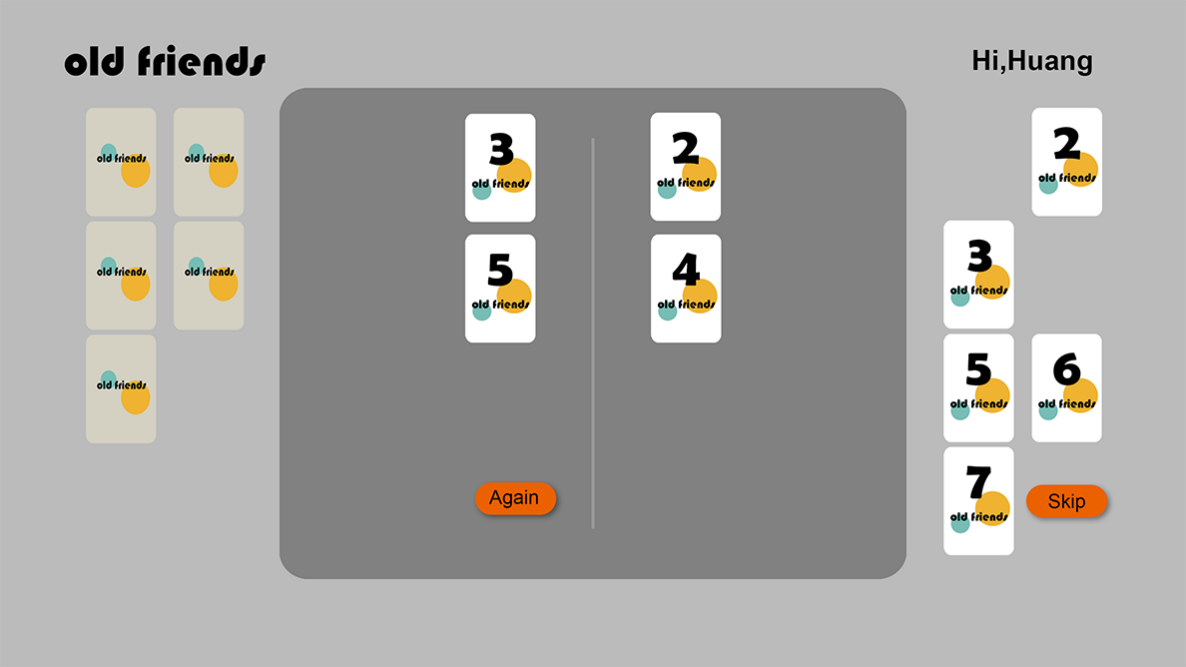
Main gameplay interface of the “Old Friends” serious card game.

The design of the game’s main interface (Figure 4) continues the aesthetic and functional principles established on the login page, aiming to create a low-stimulation environment for extended gameplay. We chose a neutral gray background, forming a calm and professional base color scheme that effectively reduces visual fatigue, adhering to the “minimum visual load principle.” Against this background, key interactive components, such as white cards and orange function buttons, are presented with high contrast to ensure immediate visual recognition and readability. The interface layout is carefully planned to guide the user’s interaction flow, with the left panel displaying the opponent’s hand, while the right area is dedicated to displaying the user’s hand, creating a clear and intuitive game cycle. The “Again” and “Skip” buttons at the bottom provide core operational controls, with their orange color ensuring high visibility. We clearly defined their functions, “Again” for haptic recognition of both players’ cards again, and “Skip” for skipping the current round, thus supporting the user’s cognitive rhythm and giving them clear operational autonomy. Emotional design remains indispensable at this stage. The consistently displayed “Old Friends” branding and personalized welcome messages, such as “Hello, Mr. Huang,” are not mere decorations. They are thoughtfully designed features to reinforce a sense of familiarity and social presence. This consistent emotional framework is crucial for aligning the core gameplay with the overall research objectives of “emotional support” and “social interaction.”

**Figure 5:**
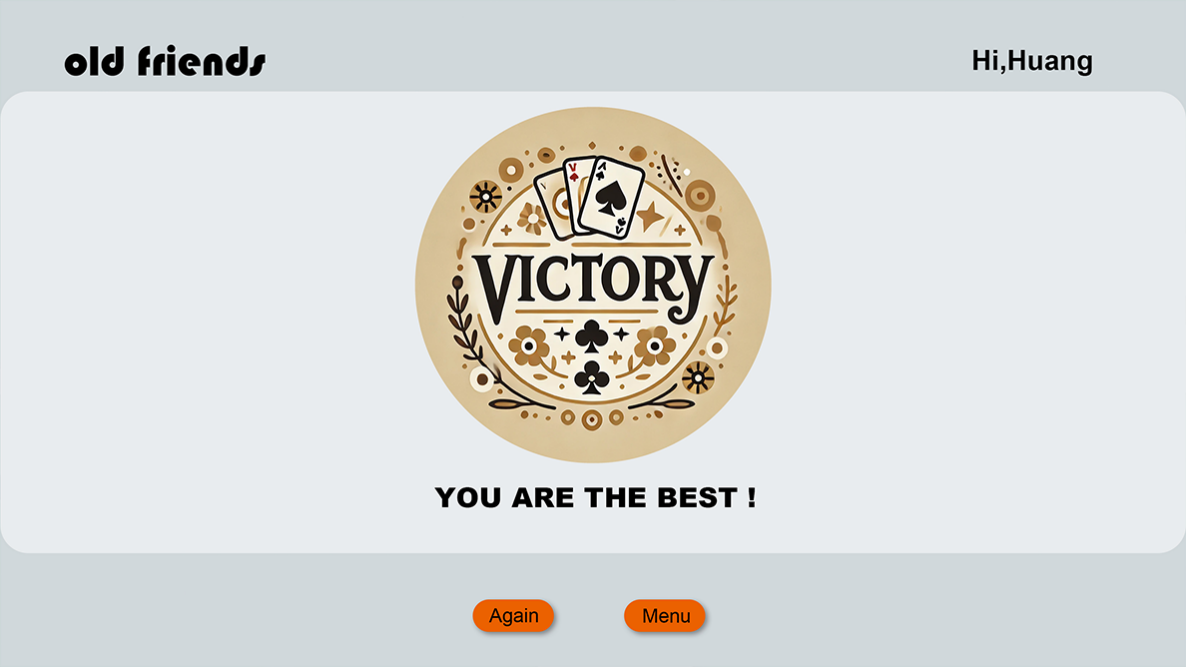
Victory outcome interface of the “Old Friends” serious card game.

**Figure 6:**
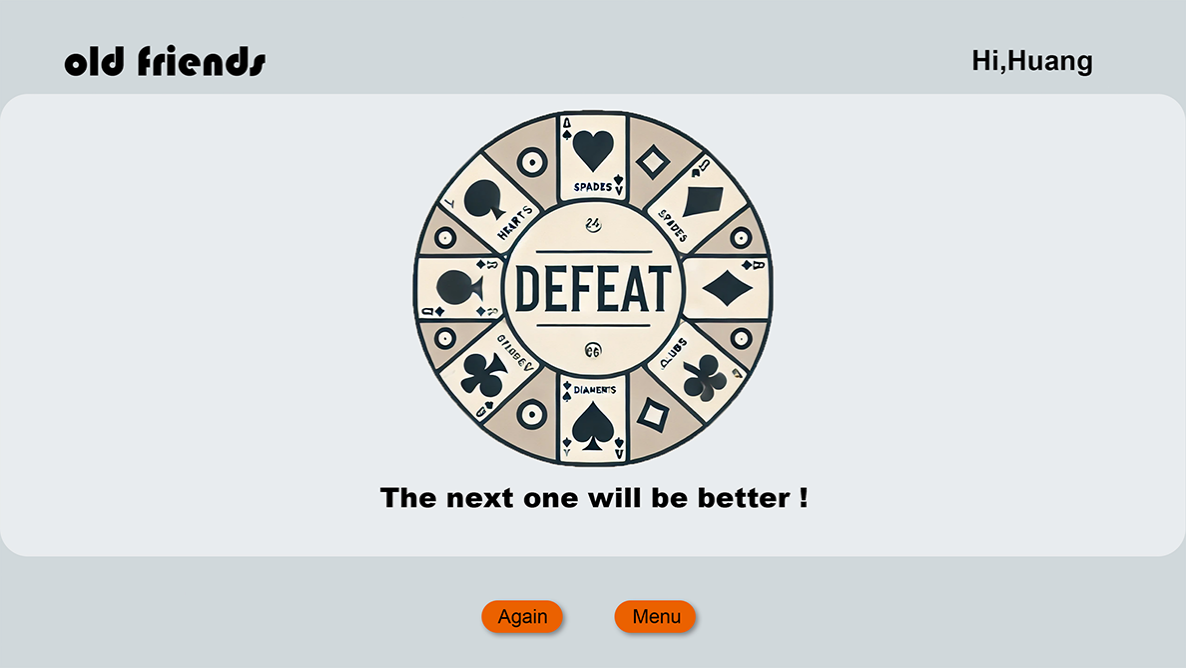
Failure outcome interface of the “Old Friends” serious card game.

The core design of the victory and defeat interfaces (Figures 5 and 6) is to provide emotionally intelligent feedback for older users. Both interfaces strictly adhere to the design language of the main interface, using a low-visual-load gray background and high-contrast text to ensure absolute clarity of information. The key design difference lies in the refined tuning of emotional semantics. The victory interface uses strongly affirmative language, such as “YOU ARE THE BEST!” to maximize the user’s sense of accomplishment and self-efficacy, while the defeat interface uses encouraging language, such as “The next one will be better!” to buffer frustration and guide the outcome toward a positive and constructive direction. This differentiated copywriting strategy is a conscious design choice to achieve the goal of “emotional support.” At the interaction level, the “Again” button is placed in the exact same position on both interfaces, following the principle of consistency. This allows users to quickly start a new game without having to search for it again, regardless of the outcome, reducing cognitive load and operational hesitation. The brand logo and personalized greeting (“Hi, Huang”) at the top serve as a consistent emotional thread, continuously reinforcing the game’s social sense of belonging. Overall, these 2 interfaces are not only the end point of the game process but also key nodes that connect the beginning and the end, maintaining the user’s emotional investment and continued participation.

**Figure 7:**
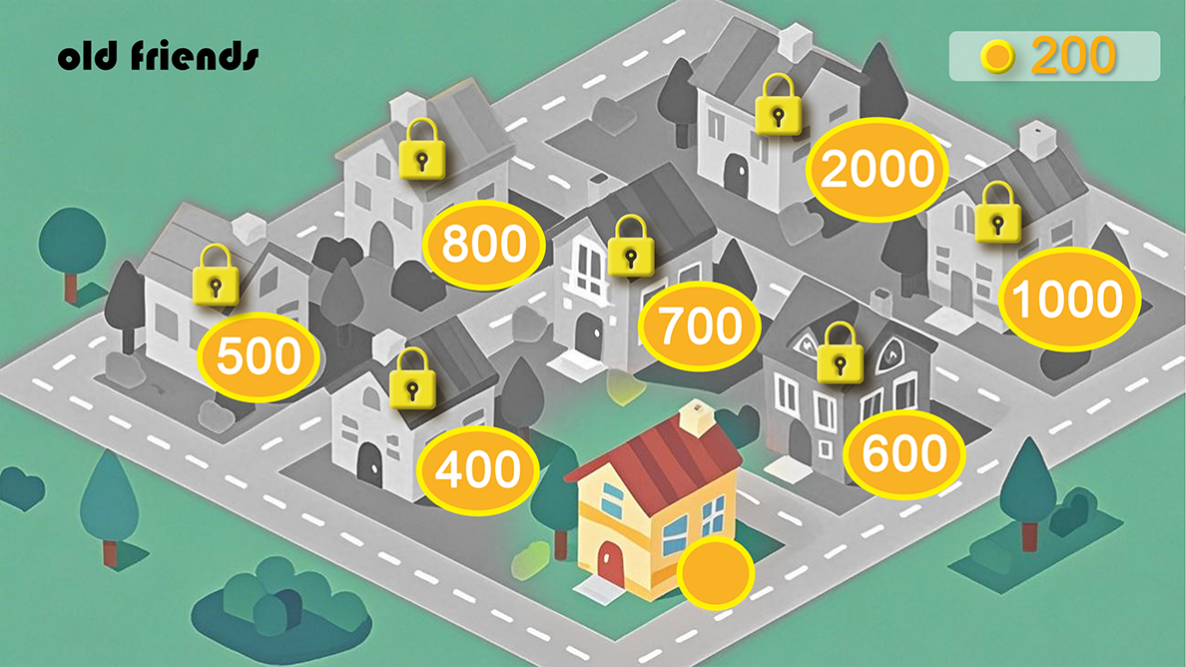
"Homeland" interface within the "Old Friends" serious card game.

The “Homeland” interface (Figure 7), serving as the core social and progress hub of the game, is designed to transform the abstract sense of community belonging into a concrete visual expression. The interface continues the game’s overall principle of low visual load, using soft backgrounds and high contrast numbers to ensure clear readability. Its core design feature is a series of circular blocks labeled with numbers. We chose circles because their smooth, rounded shape conveys a sense of inclusivity and harmony, perfectly aligning with the “Homeland” theme. The numbers within each block represent virtual coins needed for community building. This quantified display makes the abstract goal of “community building” concrete and controllable, thereby enhancing the clarity of tasks and operational confidence for older users. Overall, this interface is not merely a collection of functions but also a visual metaphor, as breaking down the “community” into interactive and achievable units aims to strengthen older users’ sense of participation and belonging, directly supporting the study’s core objectives of “social interaction” and “emotional support.”

### Haptic Interaction Flowchart

**Figure 8:**
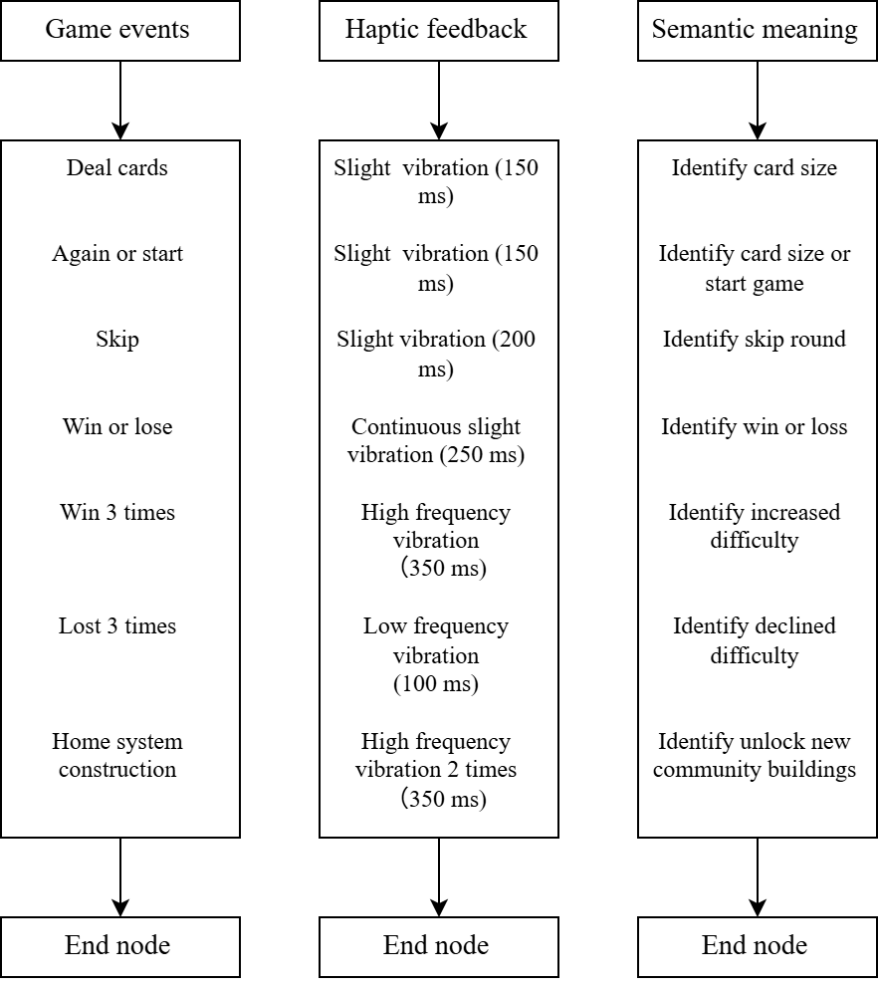
Mapping of game events to structured haptic feedback patterns in the "Old Friends" serious card game.

To create an accessible and semantically rich gaming experience for older users, we designed a structured haptic feedback system that maps specific vibration patterns to in-game events, as shown in Figure 8. This design follows a core principle of using noninvasive haptic cues to convey game status information, thereby reducing reliance on vision and providing support for users with visual impairments.

The mapping relationship is carefully designed to ensure intuitiveness. Slight vibration (150 ms) is used for “Deal cards” and pressing the “Again” and “Start” buttons. Conversely, a continuous slight vibration (250 ms) is dedicated to important game outcomes (win or loss), providing a more significant and emotionally resonant cue. Furthermore, the system dynamically adjusts the game difficulty based on player performance, conveying this through differentiated vibrations, with a high-frequency vibration (350 ms) after 3 consecutive wins indicating an increased challenge, while a low-frequency vibration (100 ms) after 3 consecutive losses indicates a decreased difficulty. Unlocking new community buildings is achieved with 2 high-frequency vibrations (350 ms each). This closed-loop design aims to maintain an appropriate level of challenge and alleviate player frustration.

In terms of technical implementation, this haptic solution uses an Android linear resonant motor, chosen for its ability to accurately and stably generate predetermined vibration parameters. Therefore, this structured approach ensures that haptic feedback is not merely decorative but serves as an indispensable and meaningful communication channel within the game’s accessibility design framework.

## Discussion

### Overview of Findings in Relation to Objectives

This pilot study successfully achieved the 2 main objectives outlined in the introduction. First, it systematically applied the DPE framework to design a haptic-driven prototype of serious game theory for older adults. Second, it conducted a preliminary assessment of the prototype’s usability, accessibility, and user experience in the target population. The results confirmed the feasibility and preliminary acceptability of the approach. Quantitative analysis showed that the prototype achieved an excellent SUS score. In the qualitative analysis, thematic analysis revealed three key user experience themes: (1) the intuitiveness of haptic feedback, (2) its effectiveness in reducing perceived eye strain, and (3) the appeal of simplified game mechanics. These results collectively provide fundamental validation that DPE-guided design with haptic feedback as a core component can create an engaging and visually low-load cognitive training tool for older adults, thus directly achieving the objectives of this study.

### Comparisons to Existing Literature

This study addresses a specific gap in the existing literature. While previous studies have successfully integrated haptic technology into serious games, their main focus has often been on enhancing realism, immersion, or motor skill training [17,19]. In contrast, our app is inherently accessibility-driven. We repurpose a common hardware feature of smartphones (vibration motors) to address a key and often overlooked problem of the pervasive visual dependence in tools designed for older populations [14,15]. Furthermore, by adopting the DPE framework, we provide a systematic, theory-based design approach that goes beyond the ad hoc or technology-centric design approaches seen in some previous work [18,20], thus providing a replicable model for future research and development in the field of inclusive aging technologies.

### Interpretations

Positive user feedback regarding the intuitiveness of haptic feedback, its reduction of eye strain, and the game’s ease of use validated our design approach in several ways. First, users quickly grasped the ability to identify cards through vibration cues, demonstrating that the carefully designed haptic symbol system successfully served as an effective alternative to traditional information channels. This directly confirms the feasibility of “haptic feedback as a substitute for vision” as a practical design principle [17,19]. Second, user feedback on reduced eye strain indicates that the strategy of combining high-contrast visuals with haptic cues successfully alleviated the burden on the visual system [15,32]. Finally, user appreciation for the simplified card mechanics suggests that for our target user group, reducing the cognitive burden associated with complex rules is just as important as sensory adaptation [33], ensuring user engagement and minimizing frustration.

### Implications

These findings are significant. Theoretically, they provide concrete empirical support for applying sensory substitution [34] and cognitive load theories [35] to the design of technology for older adults. Our research shows that information can be strategically redistributed across different sensory channels to create a more accessible and less burdensome user experience for visually impaired older adults. Practically, the results validate the DPE framework as a feasible blueprint for building such designs. The “design” dimension ensures the learnability of haptic symbols, the “play” dimension uses these symbols to implement core mechanisms, such as difficulty adjustment, and the “experience” dimension uses them to provide emotional feedback. This provides developers with a clear path to create serious games that are not only cognitively stimulating but also inherently accessible, going beyond the practice of adding accessibility features after the fact.

### Limitations

As a pilot study, this research has several limitations that must be explained to better understand the findings and guide future research. First, method limitations are significant, as the small sample size (n=10) and recruitment from a single old-age university solely through convenience sampling limit statistical power and the general applicability of the results. Second, reliance on self-reported measurements (eg, perceptions of eye fatigue during interviews) and the lack of objective physiological or cognitive indicators mean that while the reported benefits are encouraging, they remain preliminary and subjective. Third, the nature of the intervention itself also presents limitations, as a cross-sectional study design cannot assess long-term engagement, cognitive maintenance effects, or changes in the potential haptic learning curve over time. Furthermore, the technical parameters of the haptic feedback (eg, vibration pattern and duration) are based on the initial design choices and require systematic optimization.

To overcome these specific limitations, future research should prioritize the following directions: (1) conducting large-scale randomized controlled trials, including diverse populations with clinical characteristics, to verify cognitive effects using statistical methods and incorporate objective indicators; (2) using longitudinal study designs to assess users’ sustained engagement, long-term adherence, and the trajectory of cognitive outcomes; (3) systematically testing haptic parameters to establish evidence-based intuitive and emotionally resonant guidelines for older adults; and (4) expanding the prototype by incorporating richer social interaction mechanisms and adaptive narratives to further enhance long-term motivation. Ultimately, transforming this framework into standardized design guidelines will be key to advancing the field of accessible cognitive training.

### Conclusion

This pilot study validated a prototype of a haptic-driven serious game for older adults. The main innovation of this study lies in the systematic application of the DPE framework, putting “haptic as a substitute for vision” as a core design principle into practice. This approach fundamentally differs from existing studies, which largely use haptics to enhance overall immersion rather than addressing specific accessibility challenges posed by visual impairments. The main contribution of this study to the field is providing a reusable and theoretically sound design blueprint, offering a structured approach to creating efficient and easy-to-use cognitive training tools. Its implications in the real world, the prototype demonstrates a practical and scalable model for deploying low-visual-load interventions in communities and care facilities. These findings have broader implications, as they point to a more inclusive digital health future where therapeutic tools can be designed with sensory impairments in mind from the outset. By reducing barriers to participation, such approaches promise to promote social inclusion and support cognitive health among older adults, thereby meeting an important public health need.

## Abbreviations

DPE: Design-Play-Experience
JARS: Journal Article Reporting Standards
SUS: System Usability Scale

## Data Availability

The datasets generated or analyzed during this study are available from the corresponding author on reasonable request.

## References

[1] Ageing and health. World Health Organization.

[2] Petersen RC, Smith GE, Waring SC, Ivnik RJ, Tangalos EG, Kokmen E (1999). Mild cognitive impairment: clinical characterization and outcome. Arch Neurol.

[3] Larson EB, Yaffe K, Langa KM (2013). New insights into the dementia epidemic. N Engl J Med.

[4] Mouchaileh N, Hughes AJ (2020). Pharmacological management of Parkinson’s disease in older people. Pharmacy Practice and Res.

[5] Del Ser T, Zea M, Valentí M, Olazarán J, López-Álvarez J, Rebollo-Vázquez A, Ávila-Villanueva M, Frades B, Medina M, Fernández-Blázquez MA (2019). Effects of commonly prescribed drugs on cognition and mild cognitive impairment in healthy elderly people. J Psychopharmacol.

[6] Petersen RC, Bennett D (2005). Mild cognitive impairment: is it Alzheimer's disease or not?. J Alzheimers Dis.

[7] Yu J, Lam CL, Lee TMC (2017). White matter microstructural abnormalities in amnestic mild cognitive impairment: a meta-analysis of whole-brain and ROI-based studies. Neurosci Biobehav Rev.

[8] Dementia. World Health Organization.

[9] Umphred DA, Lazaro RT, Roller M, Burton G (2013). Neurological Rehabilitation.

[10] Parkinson disease. Merck Manual Professional Edition.

[11] González-González CS, Toledo-Delgado PA, Muñoz-Cruz V, Torres-Carrion PV (2019). Serious games for rehabilitation: gestural interaction in personalized gamified exercises through a recommender system. J Biomed Inform.

[12] Abt CC (1987). Serious Games.

[13] Benveniste S, Jouvelot P, Péquignot R, Yang HS, Malaka R, Hoshino J, Han JH (2010). The MINWii project: Renarcissization of patients suffering from Alzheimer’s disease through video game-based music therapy. Entertainment Computing - ICEC.

[14] Huang X, Ali NM, Sahrani S (2024). Evolution and future of serious game technology for older adults. Information.

[15] Farage MA, Miller KW, Ajayi F, Hutchins D (2012). Design principles to accommodate older adults. Glob J Health Sci.

[16] Konstantinidis EI, Bamparopoulos G, Bamidis PD (2017). Moving real exergaming engines on the web: the webFitForAll case study in an active and healthy ageing living lab environment. IEEE J Biomed Health Inform.

[17] Deng S, Chang J, Zhang JJ, De Gloria A (2014). A survey of haptics in serious gaming. Games and Learning Alliance.

[18] LI Y, Yoo Y, Weill-Duflos A, Cooperstock J (2021). Towards context-aware automatic haptic effect generation for home theatre environments. VRST '21: Proceedings of the 27th ACM Symposium on Virtual Reality Software and Technology.

[19] Pacheco-Barrios K, Ortega-Márquez J, Fregni F (2024). Haptic technology: exploring its underexplored clinical applications-a systematic review. Biomedicines.

[20] Deng S, Kirkby JA, Chang J, Zhang JJ (2014). Multimodality with eye tracking and haptics: a new horizon for serious games?. Int J Serious Games.

[21] Silva AJR, Restivo T, Gabriel J (2013). A serious game with a thermal haptic mouse. Int J Onl Eng.

[22] Ferdig RE (2009). Handbook of Research on Effective Electronic Gaming in Education (3 Volumes). IGI Global Scientific Publishing.

[23] Golzar J, Noor S, Tajik O (2022). Convenience sampling. Int J Educ Lang Stud.

[24] Su Zheng, Li Yinghua, Xie Ying, Huang Zhenxiao, Cheng Anqi, Zhou Xinmei, Li Jinxuan, Qin Rui, Wei Xiaowen, Liu Yi, Xia Xin, Song Qingqing, Zhao Liang, Liu Zhao, Xiao Dan, Wang Chen (2024). Acute and long COVID-19 symptoms and associated factors in the omicron-dominant period: a nationwide survey via the online platform Wenjuanxing in China. BMC Public Health.

[25] Brooke J (1996). SUS: a 'Quick and Dirty' usability scale. Usability Evaluation In Industry.

[26] Adeoye‐Olatunde OA, Olenik NL (2021). Research and scholarly methods: semi‐structured interviews. J Am Coll Clin Pharm.

[27] Bangor A, Kortum PT, Miller JT (2008). An empirical evaluation of the system usability scale. Int J Hum Comput Interact.

[28] Sampaio A, Lewis JR (2013). Quantifying the User Experience: Practical Statistics for User Research.

[29] Appelbaum M, Cooper H, Kline RB, Mayo-Wilson E, Nezu AM, Rao SM (2018). Journal article reporting standards for quantitative research in psychology: the APA publications and communications board task force report. Am Psychol.

[30] Willig C, Rogers WS (2017). The SAGE Handbook of Qualitative Research in Psychology.

[31] Luo R, Wang J, Wang Y (2023). Undergraduate students' perceptions of using videoconferencing for EFL learning: evidence from tencent meeting application. Heliyon.

[32] Web Content Accessibility Guidelines (WCAG) 2.1.

[33] Bilau I, Koo B, Fu E, Chau W, Kwon E, Yang E (2025). Visual accessibility through open shelving: impacts on cognitive load, motivation, physical activity, and user perception in older adults with mild cognitive impairment. J Aging Environ.

[34] Bach-y-Rita P, Kercel SW (2003). Sensory substitution and the human-machine interface. Trends Cogn Sci.

[35] (2011). Cognitive load theory. Psychology of Learning and Motivation.

